# Förster Resonance Energy Transfer between Core/Shell Quantum Dots and Bacteriorhodopsin

**DOI:** 10.1155/2012/910707

**Published:** 2012-06-10

**Authors:** Mark H. Griep, Eric M. Winder, Donald R. Lueking, Gregory A. Garrett, Shashi P. Karna, Craig R. Friedrich

**Affiliations:** ^1^Department of Mechanical Engineering Mechanics, Michigan Technological University, 815 RL Smith, 1400 Townsend Drive, Houghton, MI 49931, USA; ^2^Multi-Scale Technologies Institute, Michigan Technological University, 815 RL Smith, 1400 Townsend Drive, Houghton, MI 49931, USA; ^3^WMRD, US Army Research Laboratory, 4600 Deercreek Loop, Aberdeen Proving Ground, Adelphi, MD 21005, USA; ^4^Department of Biological Sciences, Michigan Technological University, 815 RL Smith, 1400 Townsend Drive, Houghton, MI 49931, USA; ^5^SEDD, US Army Research Laboratory, AMSRD-ARL-SE-EM, 2800 Powder Mill Road, Adelphi, MD 20783, USA

## Abstract

An energy transfer relationship between core-shell CdSe/ZnS quantum dots (QDs) and the optical protein bacteriorhodopsin (bR) is shown, demonstrating a distance-dependent energy transfer with 88.2% and 51.1% of the QD energy being transferred to the bR monomer at separation distances of 3.5 nm and 8.5 nm, respectively. Fluorescence lifetime measurements isolate nonradiative energy transfer, other than optical absorptive mechanisms, with the effective QD excited state lifetime reducing from 18.0 ns to 13.3 ns with bR integration, demonstrating the Förster resonance energy transfer contributes to 26.1% of the transferred QD energy at the 3.5 nm separation distance. The established direct energy transfer mechanism holds the potential to enhance the bR spectral range and sensitivity of energies that the protein can utilize, increasing its subsequent photocurrent generation, a significant potential expansion of the applicability of bR in solar cell, biosensing, biocomputing, optoelectronic, and imaging technologies.

## 1. Introduction

Integrated nano biosystems are expected to offer applications in multiple technologies, such as biodetection and sensing [1, 2], biomedical diagnostics [3], single molecule dynamics [4], and photovoltaics [5]. In this work, the fundamental properties of such multifunctional hybrid nano biosystems involving core-shell quantum dots (QDs) and the optical protein bacteriorhodopsin (bR) are presented.

Bacteriorhodopsin has been a subject of intense study over the past four decades due to its photoconducting properties and exceptionally high long-term stability against thermal, chemical, and photochemical degradation [6–8]. As a retinal protein found in the cell membrane of the extremophile *Halobacterium salinarum*, it is utilized to generate a proton motive force that energizes ATP synthase to drive the conversion of ADP and P_i_ to ATP and H_2_O, thereby providing the energy to drive the cell's internal machinery [9]. The proton motive force is achieved when bR's attached retinal chromophore absorbs photons in the 570 nm region, resulting in a *cis-trans* isomerization of the retinal. This structural alteration initiates proton transport from the retinal region to the extracellular side of the membrane creating a proton gradient within the membrane, with subsequent reprotonation from the cytoplasm [10]. This proton gradient across the cell membrane, which facilitates ATP synthesis in living systems, can be utilized to produce a measurable electrical response in engineered applications. Applications of bR require it to be extracted from the *H. salinarum* bacterial system, which results in purified purple membrane (PM) fragments, on average 470 nm in diameter, which are composed of multiple bR molecules and their associated lipids. With its high stability in extreme conditions and functional lifespan of several years in both wet and dried states [11], bR (differential photocurrent) has been previously utilized in a wide array of applications including photoimaging [12], light-sensitive alarm devices [13], solar cells [14–16], and holographic memory [17]. 

One potential hindrance to the broad application of bR in engineered devices is its relatively limited spectral activation range. Here, this issue is addressed by coupling bR to inorganic QDs capable of capturing a broader spectral range and transferring the captured energy directly to the bR retinal. Inorganic QDs or nanoparticles (NPs) are known to absorb photons with energy over a wide range of the spectrum from ultraviolet to the visible and exhibit bright, atom-like narrow emission bands in the visible that can be further tuned by changing the size or composition of the particles [18, 19]. Furthermore, QDs exhibit exceptionally high chemical and physical stability, low-photobleaching, and the ability to bind with multiple organic and biomolecules. Such properties have made QDs a desirable source of photons for the study of the photoassisted structural changes and dynamics of biomolecules [1, 2, 4]. Of particular significance has been the Förster resonance energy transfer (FRET) [20], a nonradiative energy transfer, process between inorganic QDs and fluorescent biomolecules [21, 22]. When inorganic QDs and biomolecules are suitably conjugated such that their spatial separation is less than 10 nm and the fluorescence emission from the QD overlaps with the absorption spectrum of the biomolecules [22, 23], the QD donor can nonradiatively transfer energy to the biomolecule acceptor. QD biomolecule FRET has been successfully used to develop and demonstrate QD-based biomolecular detection systems [1, 4, 22].

**Figure 1:**
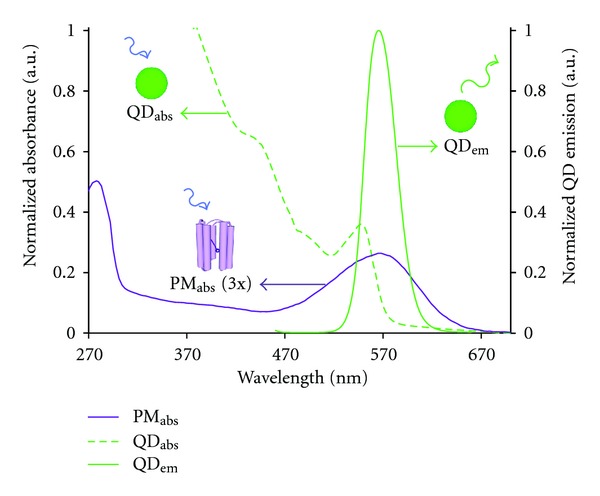
QD and bR spectra comparison at equal concentrations. QD absorption (green dashed) and emission (green solid) spectra with the bR absorption spectrum (purple solid, magnified by a factor of 3) strongly overlapping the tailored QD emission.

In the present work, we show that bR molecules and colloidal QDs together have the ability to participate in FRET coupling. The retinal molecule of bR has a strong absorption band which makes it a viable FRET acceptor [24–26], and, as shown in Figure 1, an optically tuned QD can be engineered for maximal overlap between its emission and the bR absorption spectra. For this reason, QD activation of bR via FRET has been of considerable interest in recent years [27–30]. However, previous studies could not distinguish FRET coupling between QDs and bR apart from other energy transfer processes. In the present study, we investigate the effects of QD-bR separation distances and excited state lifetime decay of QDs to establish FRET-mediated energy transfer to bR.

The spectra displayed in Figure 1 were obtained with equal concentrations of QD and PM solutions, with the 1st QD absorption peak, occurring at 544 nm, having approximately 5 times the magnitude of the bR_570 nm_ absorption peak. The QDs used in this study were 565 nm emission carboxyl-coated CdSe/ZnS (Invitrogren) core-shell QDs. An integration analysis of the absorption spectra reveals that each individual QD yields an approximate 21-fold increased photonic absorbance capacity over the bR molecule in the 270 nm–670 nm energy region. Utilizing the spectral overlap of the QD emission peak with the broad bR_570 nm_ retinal absorption region, the substantial QD energy capturing capacity can potentially be linked to the bR molecule through FRET in a properly engineered system.

## 2. Experimental Methods

### 2.1. Preparation of QD-bR Hybrid System

To achieve and maintain a constant QD-bR separation distance within the FRET coupling region, two different types of linkers were utilized to achieve two different separation distances. The shortest linkage was achieved by creating an amide bond between a QD carboxyl group (565 nm ITK Carboxyl-QD, Invitrogen) and an amine on the bR molecule. This zero-length linkage was achieved through 1-ethyl-3-(3-dimethylaminopropyl) carbodiimide hydrochloride (EDC) linker techniques. An amide linkage between the carboxyl-QD and bR amino group results in an estimated separation distance of 3 nm–5 nm between the QD core surface and the bR retinal.

Secondly, a biotin-streptavidin protocol was used to achieve an approximate 7 nm–9 nm separation distance between the QD core surface and the bR retinal. This method utilized biotinylated bR and streptavidin-coated QDs (595 nm emission, Evident Technologies). Streptavidin, a 53 kDa protein, and biotin, also known as vitamin H or B_7_, are together widely regarded as the strongest noncovalent bond in nature, with a dissociation constant (K_d_) of 4 × 10^−14^ M [31]. With its robust nature and virtually unbreakable bond, the biotin-streptavidin binding scheme has been previously used as nanoparticle linkers [32, 33]. In the present study, the biotin-streptavidin linkage was utilized to link biotinylated bR with streptavidin-coated quantum dots. The bR retinal/QD separation distance due to this bond is estimated to be ~7 nm–9 nm, assuming the streptavidin dimension of approximately 5 nm [34], 0.5 nm biotin length, QD shell thickness of 1 nm, and retinal location in the center of bR, 2 nm from the biotin/streptavidin bond.

In the present work, it was possible to biotinylate a single point on the bR molecule. Specifically, this linkage occurs on the Lysine 129 (K19) residue on the extracellular side of the protein when reacted at the proper pH [35]. It has been previously shown that bR retains its functionality after biotin attachment [36]. Thus, when streptavidin-coated quantum dots were incorporated into the system, they attached to the available biotin molecules on the bR at a single point.

To efficiently create a QD-bR linkage utilizing an EDC cross-linker, the following procedure was followed to create the QD-bR coupled pair. Initially 25 *μ*L of Invitrogen ITK Carbolxyl QD (8 nmol/L stock) was added to 60 *μ*L 2-(N-morpholino)ethanesulfonic acid (MES) buffer solution. The MES buffer was prepared to 0.1 M MES, 0.5 M NaCl at pH 5.0. Care was taken to choose a buffer that would allow for low pH stability along with remaining unreactive to the EDC molecule. In a separate vial 3 mg sulfo-NHS is added to 0.5 mL MES buffer solution, followed by the addition of 1 mg EDC. Once mixed, 25 *μ*L of the EDC/sulfo-NHS solution is added to carboxyl QDs to create amine-reactive carboxyl groups. This step is reacted for 15 minutes and is ultimately quenched by the addition of 0.2 *μ*L of 2-mercaptoethnol to quench any unreacted EDC. The nonreacted material is removed via a desalting spin column, and the MES buffer is exchanged for a 50 mM borate buffer, pH 8.3. The created amine-reactive QDs were split into two equal volumes to perform linkage reactions with both PM fragments and bR monomers and achieve a 1 : 1 QD : bR ratio. The protein is mixed with the EDC-functionalized QDs for 2 hours at room temperature to allow for maximal amide linkage formation. The unreacted amine-reactive groups are quenched by the addition of 2 uL hydroxylamine, which provides an excess of amines for binding. Finally, the reacted solution is filtered through a desalting spin column to remove the quenching agent.

### 2.2. Preparation of bR Monomers

To reduce the fragment size of the PM patches, the use of a detergent to solubilize bR monomers was used. Specifically, the detergent octyl-*β*-D-glucoside (OG) was used. The addition of OG above its critical micelle concentration (CMC) of 25 mM will allow the OG to penetrate and remove the PM lipid layer and form hydrophilic micelles with bR monomers. Over time, on the order of a day or more, the presence of OG will denature bR. The first step was to optimize the concentration of OG added to the PM solution and the amount of mixture time to provide the highest degree of protein solubilization while minimizing bR denaturation. Our analysis indicates that the optimal OG concentration ranged between 60 mM and 80 mM. These values gave the highest degree of bR solubilization with relatively low bR denaturation. The results also show that the optimal length of time to solubilize the PM ranges between 6 and 12 hours. Using an OG concentration of 70 mM, the pH of the OG solution was studied. The results for bR solubilization and denaturation with 70 mM OG over a range of pH values found that the optimal pH was 6.9 to relatively maximize solubilization with minimal bR denaturation.

## 3. Results and Discussion

Since the basic energy transfer mechanism of a QD-bR pair is unknown, the theoretical energy transfer efficiency of this hybrid system was modeled. In order to calculate the theoretical FRET efficiency of a QD-bR coupled system, the Förster radius was first determined. The Förster radius (*R*_*o*_) is the separation between the QD core and the bR retinal where 50% of the QD's energy is transferred to the bR retinal via nonphotonic energy transfer, defined as

(1) $$\begin{array}{c} R_{o}^{6}=\left(8.8\;\times \;10^{23}\;\text{mol}\right)\left(\kappa^{2}\right)\left({\eta_{D}}^{4}\right)\left(\Phi_{D}\right)\left(J\left(\lambda \right)\right), \end{array}$$

where *κ* is the dipole orientation factor (0.66 for random dipole orientation), *η*_*D*_ is the refractive index of the medium (1.33 for water), Φ_*D*_ is the quantum yield of the donor (0.62), and *J* is the normalized overlap integral between the donor and acceptor at each specific wavelength (**λ**). The *J*-integral is calculated using the (2), where *f*_*D*_ is the peak normalized fluorescence spectrum of the donor, *ε*_*A*_ is the molar absorption coefficient of the acceptor (63,000 M^−1 ^cm^−1^), and **λ** is the wavelength:

(2) $$\begin{array}{c} J=\int f_{D}\left(\lambda \right)\varepsilon_{A}\left(\lambda \right)\lambda^{4}\partial \lambda . \end{array}$$

**Figure 2:**
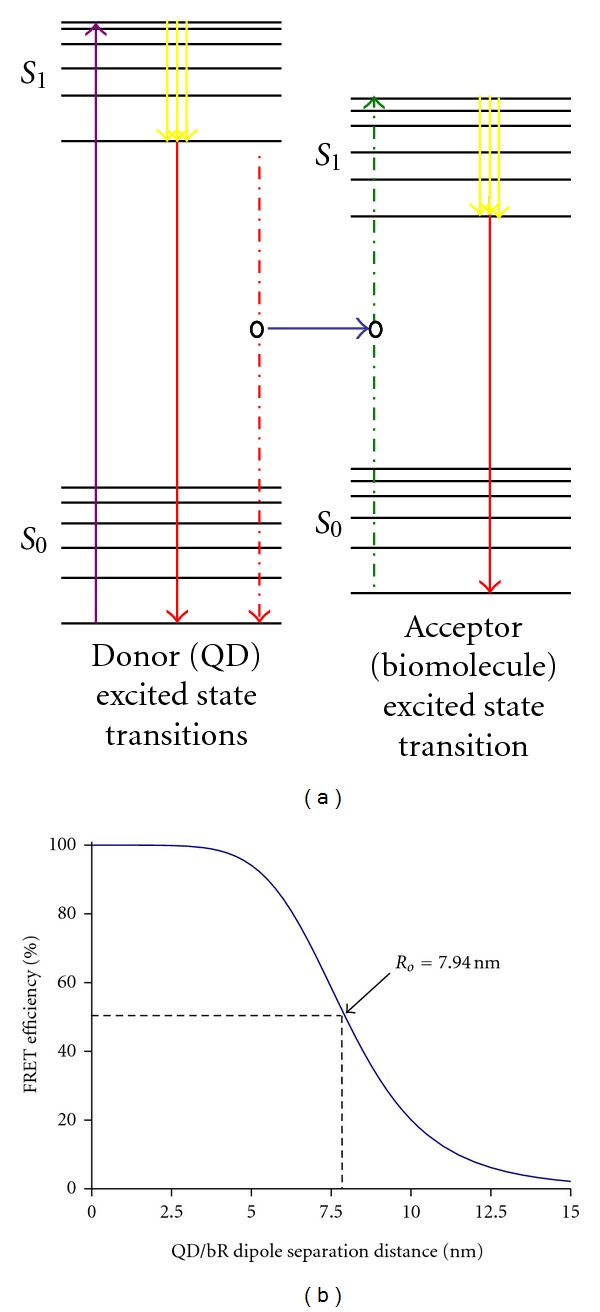
(a) Jablonski diagram showing FRET between a donor and an acceptor molecule. The purple arrow shows QD absorption, yellow arrow shows vibrational relaxation, and red solid arrow shows fluorescence. Solid blue arrow shows nonradiative energy transfer from the donor QD to acceptor biomolecule. (b) Theoretical FRET efficiency of a 565 nm emission QD (donor)-bR (acceptor) pair over a 0 nm–15 nm dipole separation range. The Förster radius of the QD-bR coupling system is calculated to be 7.94 nm.

Using these equations, *R*_*o*_ is calculated to be 7.94 nm for a 565 nm QD/bR system and 7.76 nm for a 595 nm QD/bR system. Thus, with a separation of 7.94 nm between the QD and bR retinal molecule, half of the QD output energy should be transferred to the bR molecule nonphotonically through the energy transfer process depicted in Figure 2(a). The theoretical calculations also suggest that adjusting the QD emission peak from 565 nm to 595 nm only decreases *R*_*o*_ by 0.19 nm; therefore, the use of QDs with an emission peak directly at 570 nm is not critical. With the Förster radius values determined, the theoretical FRET efficiency (E) at varying QD-bR separation distances can be determined using (3) and is plotted in Figure 2(b):

(3) $$\begin{array}{c} E=\frac{{R_{o}}^{6}}{{R_{o}}^{6}+R^{6}} \end{array}.$$

Here, a QD serves as the energy “donor” and a bR molecule serves as the energy “acceptor.” This nonradiative energy transfer, from a QD to a biomolecule, reduces (quenches) the fluorescence intensity of the QD and results in photoinduced changes, such as conformational change, proton release, and new binding events in the “acceptor” biomolecules that can be further utilized for sensing, detection, or a source of photocurrent. As seen from Figure 2(b), the energy transfer of a QD-bR hybrid can be altered substantially with subnanometer separation changes around the Förster radius. Thus, the optoelectronic properties of bR could potentially vary greatly through control of QD-bR separation to meet a specified criterion.

**Figure 3:**
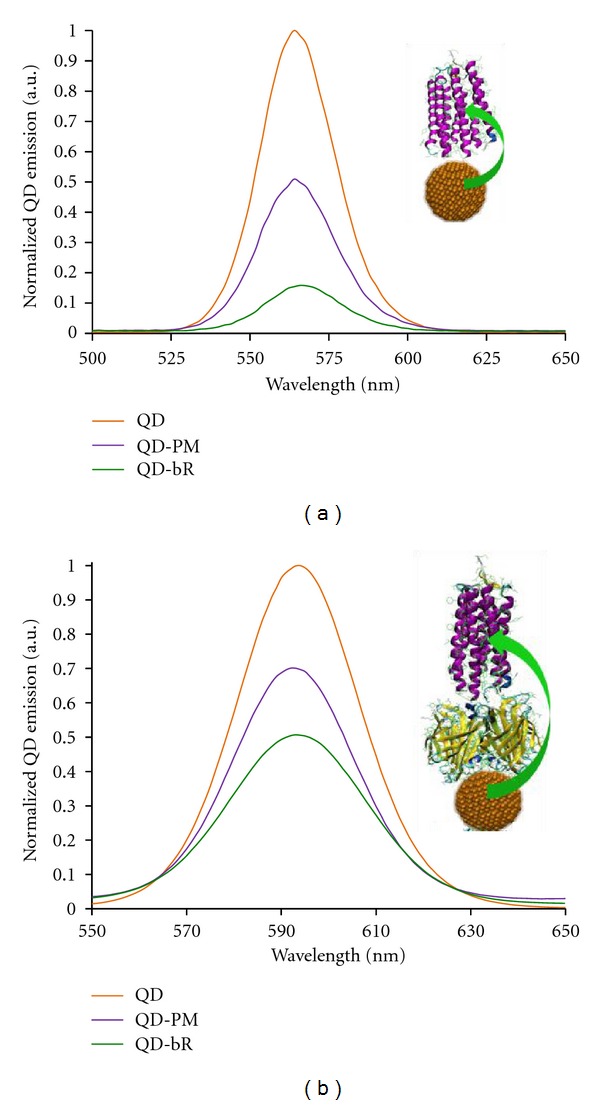
QD quenching effects of bR (PM patch and bR monomer forms) when linked to a CdSe/ZnS QD via (a) EDC and (b) biotin/streptavidin binding scheme. Inset images illustrate each linkage and estimate the QD-bR retinal separation distance to be 3.5 nm and 8.5 nm for the EDC and biotin/streptavidin linkages, respectively.

This study utilizes both short (EDC) and long (biotin/streptavidin) linking schemes to ensure QD-bR nanoscale proximity. At these varied separation distances, the energy coupling relationship between QDs and bR, in both the purple membrane fragment and bR monomer form, was analyzed. The QD quenching effects of each bR form at the given separation distance is shown in Figure 3.

The QD quenching phenomena shown in Figure 3 demonstrate energy transfer to the bR retinal from the excited CdSe/ZnS QD through both radiative and nonradiative mechanisms. In Figure 3(a), the “zero-length” EDC linker was applied to bring the QD and bR molecule into a constant 3 nm–5 nm separation distance. With the achieved bR-QD separation distance a 52%, reduction in QD emission was observed when linked to bR in the purple membrane patch form, which is due to both photonic (photon absorption) and nonphotonic energy transfer events. The observed 52% nonphotonic energy transfer is far less than the predicted value at the set separation distance, which is likely due to bR being applied in its native PM patch form. The PM patch containing the bR and its associated lipids can be considered a macromolecule, with a diameter of ~470 nm, which will fold and conform to its lowest energy state in the aqueous solution. The folding of this macromolecule likely results in many bR molecules being inaccessible to the QDs, ultimately limiting the number of bR molecules available for linkage to the QD, and thereby reducing energy transfer efficiency.

To facilitate maximal QD-bR linkage, a lipid-removal process was performed on the PM patch to isolate individual bR monomers. Specifically, the detergent octyl-B-D-glucoside (OG) was used to delipidate the PM. With bR in its monomeric form, it was attached to the QD with the aforementioned EDC linkage protocol. As shown in Figure 3(a), the resulting energy transfer efficiency of the EDC-linked QD-bR monomer hybrid system was 88.2%, demonstrating a substantial increase in QD quenching resulting from bR monomer proximity. The high degree of QD energy coupling at this short bR-QD separation more closely matches the theoretical FRET calculations in Figure 2(b); however, the actual FRET contribution versus traditional photon absorption processes is not isolated from this analysis.

As FRET is based on the inverse-sixth power of donor/acceptor separation distance, adjusting the QD-bR proximity should have a profound effect on energy transfer. We demonstrate this distance-dependent phenomenon by implementing the biotin/streptavidin linkage to increase the estimated QD-bR retinal separation distance to 7 nm–9 nm. As shown by the QD emission spectrum in Figure 3(b), the linkage of QDs to bR in the PM patch form results in a QD photonic emission reduction to 32.6% energy transfer efficiency. As with the EDC linkage, biotinylated bR in the monomeric form was also linked to the streptavidin-coated QDs and resulted in enhanced QD quenching. The greater accessibility of QDs to the bR monomers resulted in an energy transfer efficiency of 51.1%.

**Figure 4:**
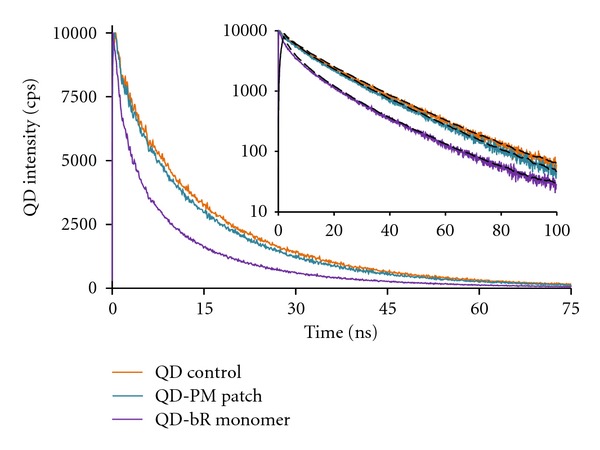
QD lifetimes when linked to bR in PM patch form and bR monomer form compared to the QD only control. Inset displays identical QD excited state lifetime counts in Log-scale with theoretical fitting (dashed).

In order to establish a FRET coupling relationship between the QD core and bR retinal that is involved in the QD emission reduction, as opposed to other absorptive or concentration effects, the excited state lifetime of the QD was measured. Utilizing a 100fs laser excitation pulse with a 25 ps resolution detection technique, the QD electrons were excited and the electron-hole recombination rates were measured. In a FRET-coupled system, a portion of the excited electron energy will transfer to the overlapping energy band in the acceptor molecule, thus reducing the amount of QD photons released over time and ultimately reducing the QD excited state lifetime. With this technique, the energy transfer relationship can be isolated from other quenching phenomenon and concentration effects. The fluorescence lifetimes were measured at the QD emission maximum of 565 nm and monitored the electron energy transfer to bR, in both monomeric and PM fragment form, when directly linked via a zero-length EDC linker. The wavelength of the excitation laser was set to 340 nm to minimize activation of the bR photoresponse.  Figure 4 shows the excited state decay spectra of QD, QD-PM, and QD-bR monomer systems.

The spectra display multiexponential decays, with the carboxyl-QD control group yielding excited state lifetimes of *τ*_QD1_ = 4.7 ns (8.3%), *τ*_QD2_ = 17.9 ns (83.6%), and *τ*_QD3_ = 58.9 ns (8.1%), the percentage values indicate proportion of total lifetime contribution. The overall effective lifetime of the QD excited state was calculated to be *τ*_QDeff_ = 18.0 ns (*χ* ^2^ = 1.195). Energy transfer systems based on core-shell QD donors typically display biexponential decays with fast (several nanoseconds) and slow (tens of nanoseconds) components. Multiple theories for the origins of the fast/slow decay channels exist, suggesting delocalized/localized carriers [37] or core/surface states [38]. The best fit lifetimes for the QD control was found to correspond to three different lifetime channels, with the third channel likely due to electron trapping in the QD functionalization layer [39].

With the introduction of chemically linked PM patches, resulting in an approximate 3 nm–5 nm bR retinal-QD separation, the QD lifetimes reduce to *τ*_H-PM1_ = 4.2 ns (9.2%), *τ*_H-PM2_ = 17.0 ns (82.5%), and *τ*_H-PM3_ = 49.7 ns (8.3%), with the H subscript representing “QD-Protein Hybrid.” Concurrent with traditional fluorescence measurements, shown in Figure 3(a), the linking of bR monomers to the QD substantially increases the coupling effect, resulting in a QD lifetimes of *τ*_H-bR1_ = 1.3 ns (5.2%), *τ*_H-bR2_ = 5.6 ns (25.8%), *τ*_H-bR3_ = 16.9 ns (62.8%), and *τ*_H-bR4_ = 66.1 ns (7.2%). A possible additional slow decay channel, not apparent in the QD-PM hybrid, is seen in the QD-bR monomer fit. The overall effective lifetime of the QD-PM and QD-bR linked systems was determined to be *τ*_H-PMeff_ = 16.8 ns (*χ* ^2^ = 1.166) and *τ*_H-bReff_ = 13.3 ns (*χ* ^2^ = 1.114), suggesting energy transfer efficiencies of 6.7% and 26.1% for the QD-PM and QD-bR complexes, respectively. The FRET efficiency values, when compared to the overall QD quenching in the QD-bR hybrid system, accounts for 12.9% and 29.7% of the transferred energy in the QD-PM and QD-bR monomer systems, respectively. The greater degree of FRET coupling in the bR monomer system is expected due to increased access/binding potential of the QDs to the bR molecules.

Unlike many traditional QD-bio FRET coupled systems, the acceptor molecule bR in the present study does not utilize the transferred energy for photon generation. Instead, the bR likely dissipates this energy through both vibrations and conformational changes associated with the bR photocycle. The observed multiexponential decay timeline suggests variation of QD decay dynamics with the incorporation of bR due to the relative increase of the slower QD lifetime components. In the QD-PM hybrid, the additional decay channel is potentially due to the transfer of fast excitonic energy from the excited QD to the bR retinal, rapidly initiating the bR photocycle and altering the bR absorption in such a way that likely reduces the efficiency for further energy transfer. Further studies will evaluate if the coupled QD FRET energy contributes to enhancing the bR photoelectric output.

## 4. Conclusion

In summary, FRET between CdSe/ZnS core-shell QDs and bR has been demonstrated. Advantage is taken of the bR 570 nm absorption band overlap and the intense energy emission of engineered QDs. Enhanced FRET efficiency is observed when QDs are coupled directly to bR monomers rather than the PM fragments containing bR and its associated lipids, likely due to the absence of steric hindrances and limited protein availability due to membrane folding in the control PM fragments, ultimately facilitating greater bR-QD interactions. Further, directly bound QD-bR hybrids via EDC show greater energy transfer efficiency than biotin-streptavidin linked QD-bR conjugates, demonstrating the distance-dependent nature of this FRET coupled system.
